# From Food Additives and Contaminants to the Human Exposome: A Precision Toxicology Framework for Next-Generation Food Safety

**DOI:** 10.3390/foods15193525

**Published:** 2026-10-02

**Authors:** Mohamed M. Eltanty, Jae-Han Shim, Hasan Türkez, Su-Jin Bae, Jaehoon Bae, A. M. Abd El-Aty

**Affiliations:** 1 Department of Food Hygiene and Control, Faculty of Veterinary Medicine, King Salman International University, El-Tor 46618, Egypt; muhamed.mostafa@ksiu.edu.eg; 2 Natural Products Chemistry Laboratory, College of Agriculture and Life Sciences, Chonnam National University, Gwangju 61186, Republic of Korea; jhshim@jnu.ac.kr; 3 Department of Medical Biology, Faculty of Medicine, Atatürk University, Erzurum 25240, Türkiye; 4 Functional Food Research Institute, Industry–Academic Cooperation Foundation, Daegu Haany University, Gyeongsan 38610, Republic of Korea; realsujin@dhu.ac.kr; 5 Department of Food Industry, Daegu Haany University, Gyeongsan 38610, Republic of Korea; 6 Department of Pharmacology, Faculty of Veterinary Medicine, Cairo University, Giza 12211, Egypt; 7 Department of Medical Pharmacology, Faculty of Medicine, Atatürk University, Erzurum 25240, Türkiye

**Keywords:** precision toxicology, dietary exposome, exposomics, food contaminants, human biomonitoring, food safety, chemical mixture risk assessment, life-course exposure

## Abstract

Human exposure to foodborne xenobiotics, including environmental contaminants that enter the food chain, is no longer adequately represented by isolated chemicals. Individuals encounter complex and dynamic mixtures of food additives, pesticide residues, veterinary drugs, mycotoxins, processing contaminants, packaging migrants, environmental pollutants, and other dietary chemicals that interact with genetics, metabolism, the gut microbiome, nutritional status, and lifestyle throughout life. The dietary exposome therefore represents a critical interface linking food contaminants, internal chemical exposure, molecular perturbation, and long-term human health. Conventional toxicology provides an indispensable foundation for food safety regulation through hazard characterization and population-based health guidance values but is less equipped to explain interindividual variability in biological response and chronic disease susceptibility. Precision toxicology provides an integrative and mechanism-informed framework that combines high-resolution analytical chemistry, human biomonitoring, exposomics, multiomics, new approach methodologies, and artificial intelligence. This integration enables the characterization of biologically effective exposure, the elucidation of exposure–response relationships, and the identification of interindividual variability in toxicological susceptibility. This perspective proposes a translational framework connecting external exposure → internal dose → biological response → individual susceptibility → health outcome. We position this framework relative to existing exposome, exposure-pathway, and adverse-outcome-pathway frameworks; specify the evidence required at each transition; make its life-course dimension explicit; illustrate it with a worked per- and polyfluoroalkyl substance (PFAS) example; and outline a stepwise route into food risk assessment together with its principal limitations. Such integration could advance food safety science beyond residue detection toward biologically informed exposure assessment, ultimately supporting more evidence-based regulatory decision-making, preventive strategies, and public health protection.

## 1. Introduction

For decades, food safety toxicology has largely operated under a reductionist framework in which individual chemicals are evaluated according to their occurrence, estimated intake, toxicological properties, dose–response relationships, and established health-based guidance values [1,2]. This approach has been fundamental to modern food safety regulation [3]. Humans have always been exposed to a wide spectrum of natural and anthropogenic chemicals through food; what has changed is not the presence of such exposure but its composition, the analytical resolution at which it can now be characterized, and the recognition that these substances are encountered as dynamic mixtures rather than as isolated agents. Humans are continuously exposed to chemical mixtures that enter food by fundamentally different routes; these include substances intentionally added for technological purposes, such as preservatives, colorants, sweeteners, emulsifiers, and other food additives [4,5]; residues arising from agricultural and veterinary use [1]; biological contamination, such as that resulting from the formation of mycotoxins during growth and storage, the geographical distribution of which is being reshaped by climate change [6]; chemical reactions during processing and cooking, which generate compounds, such as acrylamide and furan [7]; migration from packaging and food-contact materials [8]; and persistent environmental background contamination, including dioxins [9] and per- and polyfluoroalkyl substances (PFAS) [10]. These pathways are regulated separately and monitored through separate surveillance programs, yet all converge on the same consumer, alongside numerous lifestyle and nonchemical environmental influences. The exposome concept was introduced to capture this complexity by encompassing environmental exposures and their associated biological responses across the life course [11,12,13].

Food represents a particularly important interface between the external environment and the human exposome. Dietary exposure is chronic, heterogeneous, and influenced by geography, culture, age, socioeconomic conditions, food-processing practices, nutritional patterns, and lifestyle. The concept of the dietary exposome therefore provides an opportunity to move beyond evaluating individual contaminants toward understanding the broader chemical environment encountered through food [14]. Recent applications of exposomic workflows have already surfaced contaminants, replacement compounds, and transformation products that routine surveillance does not capture, confirming that this is now an operational capability rather than a purely conceptual one [15]. Exposure also begins before birth and varies systematically across the life course; thus, the same average annual intake can have very different biological meanings depending on when it occurs [16]. The central challenge is not only to determine which chemicals are present in food but also to establish which exposures reach humans, at what internal dose, how they perturb biological systems, and why their consequences differ among individuals. In this context, we propose an integrative precision toxicology framework, developed in Section 7, that connects what is present in food with what happens in the people who eat it. This framework provides a mechanistic and translational basis for advancing biologically informed food safety assessment and regulatory decision-making.

This article is a perspective rather than a systematic review, and its purpose is to articulate a conceptual and operational framework rather than to appraise a defined body of evidence exhaustively. Its scope is the dietary exposome, that is, the component of the human exposome delivered through food: intentionally added substances, residues of pesticides and veterinary drugs, natural toxins, process contaminants, food-contact migrants, and environmental contaminants insofar as they reach the consumer through the food chain. Nondietary routes, such as inhalation and dermal contact, are considered only when they complicate the interpretation of biomarkers of dietary exposure. The supporting literature was selected narratively through targeted searches of PubMed, Scopus, and Web of Science carried out between March and September 2026, using combinations of the terms ‘exposome’, ‘dietary exposome’, ‘precision toxicology’, ‘human biomonitoring’, ‘new approach methodologies’, ‘mixture risk assessment’, ‘adverse outcome pathway’, and ‘food contaminants’, supplemented by the guidance documents and scientific opinions of the European Food Safety Authority (EFSA), the World Health Organization (WHO), and the Joint FAO/WHO Expert Committee on Food Additives (JECFA). Priority was given to peer-reviewed studies, agency opinions, and consortium outputs published between 2023 and 2026, and earlier work was retained where it remains the primary source for a concept, a validated biomarker, or a regulatory value. The remainder of the article is organized as follows. Section 2, Section 3, Section 4 and Section 5 develop the scientific case for moving from external occurrence data to biologically effective and individually variable exposure; Section 6 considers the role and limits of artificial intelligence; Section 7 presents the framework, its relation to existing frameworks, and the evidence required at each transition; Section 8 makes its temporal dimension explicit; Section 9 applies it to perfluoroalkyl substances; Section 10 sets out a stepwise route into regulatory risk assessment; Section 11 summarizes the limitations; and Section 12 and Section 13 consider public health implications and a research and regulatory roadmap.

## 2. From Food Contamination to Internal Exposure

Advanced tandem and high-resolution mass spectrometry platforms, such as liquid chromatography–tandem mass spectrometry (LC–MS/MS), gas chromatography–tandem mass spectrometry (GC–MS/MS), quadrupole time-of-flight mass spectrometry (QTOF–MS), and Orbitrap mass spectrometry, have substantially expanded the capabilities of exposure analysis [17,18] and of food-contaminant analysis, enabling increasingly sensitive multiresidue determination in complex food matrices [19]. High-resolution suspect and nontarget screening can further reveal emerging contaminants, transformation products, and previously unrecognized exposure signatures that targeted monitoring may overlook [15]. Nevertheless, analytical detection alone does not establish toxicological relevance.

Because this distinction recurs throughout the framework, the terms used here are defined explicitly. Occurrence, as used here, denotes the presence of a chemical in food; external exposure, the amount reaching the body through the diet over a defined period; and internal exposure, or internal (absorbed) dose, the amount that crosses an absorption barrier and is present in the systemic circulation or in a biological matrix [20]. The biologically effective dose is the part of the internal dose that reaches the target tissue or molecular site in a form able to interact with it, and it may differ substantially from the concentration measured in blood or urine [21]. A biological response is any measurable change resulting from that interaction; it constitutes an adverse effect only when it impairs functional capacity or the capacity to compensate for additional stress. Hazard is the inherent potential of a chemical to cause an adverse effect, and risk is the probability of that effect occurring under specified conditions of exposure [1,20]. These are distinct levels of evidence: detecting a chemical in food establishes occurrence, not exposure; measuring it in blood establishes internal exposure, not effect; and observing a molecular perturbation establishes a biological response, not risk.

Following ingestion, absorption, distribution, metabolism, and excretion determine the biologically effective dose. Human biomonitoring provides a critical link between external exposure and internal dose by quantifying parent xenobiotics, metabolites, adducts, or other exposure-related biomarkers in blood, urine, and other biological matrices [22]. These biomarkers differ in what they capture: short-lived urinary metabolites reflect recent intake; protein or DNA adducts and persistent parent compounds in serum reflect cumulative internal dose; and biomarkers of effect, such as markers of oxidative or genotoxic damage, reflect biological response rather than exposure [21,23]. This approach is increasingly relevant not only to environmental contaminants but also to food additives and food-contact chemicals, for which biomarker-based assessment can help bridge dietary occurrence and biologically internalized exposure [24]. Future food safety assessments should increasingly integrate food occurrence data, dietary consumption, toxicokinetics, and human biomonitoring, thereby shifting the emphasis from what people are estimated to consume toward what their biological systems actually encounter. The next frontier in food safety is therefore not only to achieve more sensitive detection but also to connect external chemical occurrence with biologically effective internal exposure [25].

Two examples show the value of this approach. Aflatoxin exposure has been measured for many years using the aflatoxin B1–lysine adduct found in serum albumin. This biomarker has been validated using different analytical methods and in several population studies [23,26]. Because the adduct persists for approximately two to three months, it is a biomarker of cumulative internal dose, whereas urinary aflatoxin metabolites indicate mainly recent exposure [23]. This information cannot be obtained from food occurrence data alone.

The internal dose can also directly support health-based regulations. With respect to four perfluoroalkyl substances—perfluorooctanoic acid (PFOA), perfluorononanoic acid (PFNA), perfluorohexanesulfonic acid (PFHxS), and perfluorooctanesulfonic acid (PFOS)—EFSA identified a serum concentration of 17.5 ng/mL in one-year-old children as the benchmark dose lower confidence limit (BMDL) for reduced antibody responses to routine vaccination [27,28]. Physiologically based kinetic (PBK) modeling was subsequently used to estimate the maternal intake corresponding to this internal concentration, yielding a tolerable weekly intake of 4.4 ng/kg body weight per week for the sum of the four substances [28]. This derivation is methodologically significant because the point of departure is an internal concentration rather than an administered dose: the guidance value is anchored to what was measured in human serum, and external intake is reconstructed from it, inverting the conventional direction of risk assessment.

## 3. Beyond the Single-Chemical Paradigm

Humans are rarely exposed to one chemical at a time. Real-world mixtures may act independently, additively, synergistically, or antagonistically, and chemicals with different structures may converge on common molecular pathways or adverse outcomes [29,30]. Regulatory frameworks have consequently begun to recognize the importance of combined chemical exposure [30].

Current practice rests on two reference models. Dose addition assumes a common mode of action so that components behave as dilutions of one another scaled by their potency; response addition assumes independent modes of action so that the combined effect is derived from the probability of each component acting alone. EFSA recommends dose addition as the default assumption, unless there is evidence that response addition is more appropriate, because it is generally protective [30]. Component-based assessment, which combines individually characterized chemicals through the hazard index, relative potency factors, or the combined margin of exposure, is more widely applicable than whole-mixture testing, which requires the mixture itself to be tested and is therefore practical mainly for mixtures of stable, reproducible composition [30,31]. Components are combined within cumulative assessment groups, formed preferably on a shared mode of action or adverse outcome pathway and otherwise on a common adverse outcome or target organ, with structural similarity as a supporting criterion [32].

Precision toxicology can improve this practice in three ways. First, human biomonitoring replaces assumed combinations with measured internal exposures; a biomonitoring-based assessment of European data has identified the chemicals driving potential mixture risk directly from measured internal concentrations [33]. Second, mechanistic data allow cumulative assessment groups to be formed on shared key events rather than on structure or target organs alone, which is the basis that current grouping guidance prefers but can rarely populate [32]. Third, internal dose measurements, which correspond to the target-site exposure defined in the aggregate exposure pathway framework [25], allow dose addition to be applied to the concentrations reaching the target rather than to estimated intakes, reducing the error that arises when components differ in absorption and persistence.

Mechanistic information on which such grouping depends comes largely from adverse outcome pathways (AOPs) and new approach methodologies (NAMs), including high-throughput screening, advanced cell models, organoids, microphysiological systems, computational toxicology, and omics-based approaches, which can help determine whether analytically detected exposures produce biologically meaningful perturbations [34,35]. A major advantage of many NAMs is their ability to incorporate human-relevant biological systems and mechanistic endpoints, thereby reducing the reliance on interspecies extrapolation and enabling biological perturbations to be examined at the level of human-relevant pathways [35,36].

The term “precision toxicology” is itself used in more than one sense, and it is useful to be explicit about which is intended here. The Precision Toxicology (PrecisionTox) initiative, an international consortium, has developed the term principally in relation to hazard identification. It uses evolutionarily distant, non-mammalian model organisms—including *Drosophila melanogaster*, *Caenorhabditis elegans*, *Daphnia magna*, and the embryonic stages of *Xenopus laevis* and *Danio rerio*—together with human cell lines to identify key molecular events that are conserved across distantly related animal species and can therefore be used to classify and assess chemicals without conventional mammalian testing; that program also addresses genetic variation in individual susceptibility [37]. Our use of the term is complementary rather than competing: the consortium works on the hazard side of the equation, establishing which molecular events matter and why they are transferable between species, whereas we apply the term to the exposure side, that is, to the resolution of who is actually exposed, to what internal dose, and with what individual biological consequences.

## 4. Exposomics: Connecting Chemistry with Biology

The transformative value of exposomics lies not only in detecting more chemicals but also in connecting chemical exposure with biological response [12,38]. Combining exposomic data with genomics, epigenomics, transcriptomics, proteomics, metabolomics, lipidomics, and metagenomics enables the identification of molecular perturbations that link chemical exposure to early biological effects and potentially adverse health outcomes [12,13,39]. Single-cell multiomic mapping of the human body has shown extensive cell type-specific variation in DNA methylation and three-dimensional genome organization, highlighting the importance of cellular context when interpreting epigenomic alterations [40]. Large-scale human proteomic studies have further revealed substantial interindividual variation in circulating molecular profiles associated with age, sex, and disease state, underscoring the importance of accounting for biological heterogeneity when molecular signatures are interpreted [41]. Such mechanistic anchoring is important because food- and environment-related xenobiotics can induce measurable oxidative and genotoxic responses in human cellular systems, as demonstrated for exposure to pesticides such as permethrin [42]. Contemporary exposome science therefore provides an important bridge between analytical toxicology and systems biology [12,39].

An additional component deserving greater attention is the gut microbiome [43]. Intestinal microorganisms can transform xenobiotics into metabolites with greater, lower, or qualitatively different biological activities, whereas xenobiotic exposure itself can alter microbial ecology. Consequently, identical external exposures may not necessarily produce identical internal chemical environments in different individuals [44]. Population-level variation in the gut microbiome may also reflect long-term host–microbial interactions with specific microbial lineages showing patterns of codiversification with human populations [45]. Such host–microbiome heterogeneity represents an additional source of biological variability that should be considered in exposome-based studies. At the exposome–host interface, the microbiome may function as a bidirectional modulator of the relationship between environmental exposure and downstream biological responses.

These possibilities should be weighed against substantial limitations. The gut microbiome composition fluctuates within individuals over days to weeks [46] and is shaped by diet, medication, age, and health status [47]; thus, a single sample may not represent the microbial context in which exposure occurs. A considerable part of the variation between studies is technical, arising from differences in specimen handling, DNA extraction, and bioinformatic processing [48]. Most associations between microbial features and xenobiotic metabolism are also correlational, and causal claims rely largely on experimental models, including human microbiota-associated rodents, whose transferability to humans has itself been questioned [49]. Standardized sampling, demonstrated reproducibility across studies, and causal evidence are therefore preconditions for incorporating microbiome data into individualized risk assessment.

## 5. Population-Average Risk and Individual Susceptibility

Perhaps the most important conceptual advance offered by precision toxicology is recognition that the same external dose does not necessarily produce the same internal dose or biological response. The implication is fundamental: the analytical equivalence of exposure does not imply the biological equivalence of risk. Individuals experiencing comparable external exposures may differ substantially in internal dose and molecular response because of variations in age, biological sex, genetics, metabolism, microbiome composition, nutritional status, disease state, and coexposures. Xenobiotic-metabolizing enzymes provide a clear example. Cytochrome P450 (CYP450) activity varies severalfold among individuals as a result of genetic polymorphisms, dietary composition, disease state, concomitant medication, and prior chemical exposure [50]. Emerging exposome research suggests that these environmental influences can modify CYP450 expression and activity, creating an important intersection between food toxicology, pharmacology, and personalized medicine [51]. Because several of these determinants, including age, physiological state, diet, and medication, change over time, susceptibility is better treated as a time-varying property of an individual than as a fixed trait, a point developed in Section 8.

Conventional risk assessment accommodates population variability primarily through default uncertainty factors, which function as deliberate placeholders for data that are historically unobtainable. Precision toxicology offers a means of populating those placeholders with measurements: chemical-specific adjustment factors derived from human toxicokinetic and toxicodynamic data rather than from generic assumptions [52]. Integration of exposure biomarkers with molecular and clinical phenotypes may eventually allow the identification of population subgroups that exhibit stronger biological responses at comparable external exposure levels.

These refinements complement rather than replace population-based thresholds. Health-based guidance values protect populations precisely because they do not require knowing who is susceptible, and person-specific exposure limits would be neither enforceable nor practical. Individual variability enters regulation through the parameters of population assessment rather than through individualized limits: through the adjustment factors just described, through probabilistic hazard characterization, which states the fraction of the population a guidance value is intended to protect [53], and through guidance values set to protect an identified susceptible subgroup, as in the PFAS assessment described in Section 2, which protects breastfed infants through a limit on maternal intake [28]. Precision toxicology therefore stratifies and refines population risk assessment rather than individualizing it.

## 6. Artificial Intelligence: Powerful but Not Sufficient

The scale, heterogeneity, and high dimensionality of exposomic and multiomic datasets increasingly require advanced computational and artificial intelligence (AI)-based approaches for data integration, pattern recognition, and biological interpretation. Machine learning and AI can help integrate heterogeneous chemical and biological datasets, explore complex mixtures, and potentially predict susceptible phenotypes [54]. In food safety, machine learning has been applied to hazard prediction and to spectroscopic and imaging-based screening, although limited interpretability remains a major obstacle to its use in decision-making [55]. However, AI should not become a substitute for mechanistic toxicology. A statistically powerful algorithm that cannot explain the biological basis of a prediction has limited regulatory value. Models intended to influence health or regulatory decisions should therefore be transparent, reproducible, externally validated, and biologically interpretable [56]. AI is perhaps most valuable as a hypothesis-generation and prioritization tool that directs experimental and mechanistic investigations rather than replacing them.

These requirements can be made specific because the main threats to model validity arise at identifiable stages. At the data stage, batch effects between analytical runs or laboratories and confounding can generate apparent signals unrelated to biology, and class imbalance can inflate apparent performance [57,58]; measurement error and nonrandom missing data can further bias the associations that models learn [59,60]. At the modeling stage, overfitting and leakage of information between training and test data can inflate the reported performance [58,61], so performance should be confirmed on independent data [62]. At the deployment stage, shifts between populations, analytical platforms, and time periods can degrade performance without warning [58], so predictions should be reported with quantified uncertainty [63]. High predictive performance is therefore insufficient for regulatory adoption: a model intended to inform regulation must also have a defined endpoint, an unambiguous algorithm, a defined applicability domain, demonstrated robustness and predictivity, and, where possible, a mechanistic interpretation [62]. On the basis of these criteria, AI in toxicology should be assessed not only by predictive accuracy but also by biological interpretability, transportability across populations, and its ability to generate experimentally testable mechanisms.

## 7. A Translational Framework for Precision Toxicology

**Figure 1:**
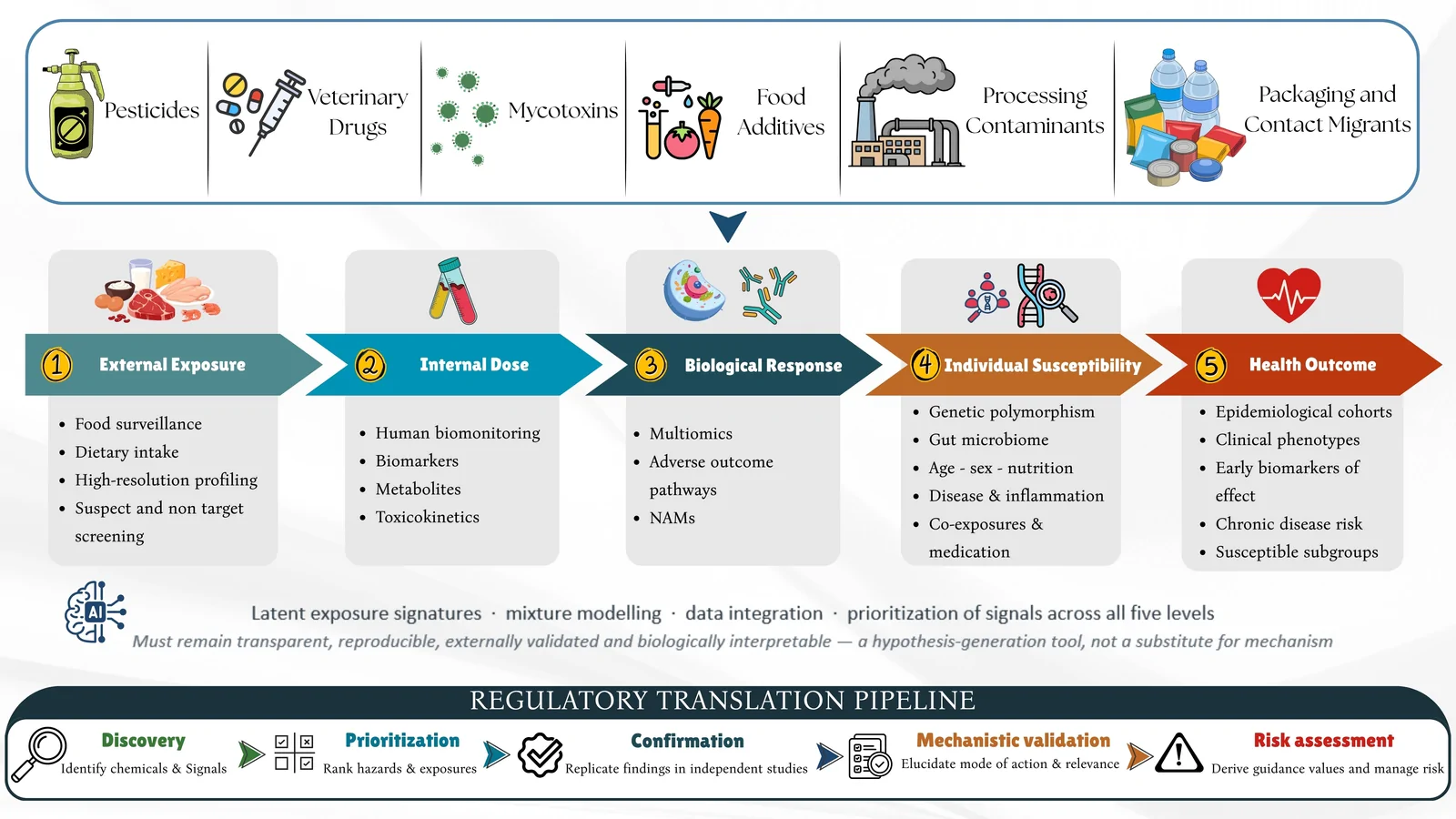
Translational precision toxicology framework for the dietary exposome. Food-derived exposures, from food additives to environmental contaminants entering the food chain, are traced through five levels—external exposure, internal dose, biological response, individual susceptibility, and health outcome—and linked to a regulatory translation pipeline (discovery → prioritization → confirmation → mechanistic validation → risk assessment). Artificial intelligence is shown as a cross-cutting layer for signal integration, mixture modeling, and prioritization across all five levels. The components that are already used in regulatory practice and the remaining research tools are presented in Section 10 and Section 11.

We propose a translational precision toxicology framework in which next-generation food safety is organized around five interconnected levels: external exposure → internal dose → biological response → individual susceptibility → health outcome (Figure 1).

At the external exposure level, food monitoring, dietary assessment, and high-resolution chemical profiling define the xenobiotics to which individuals are potentially exposed. At the internal dose level, biomonitoring and toxicokinetic information can be used to determine which chemicals and metabolites actually enter the human biological environment. At the biological response level, multiomics, mechanistic biomarkers, AOPs, and NAMs characterize early molecular perturbations. At the susceptibility level, genetics, microbiome composition, age, sex, nutrition, disease state, and coexposures explain interindividual variability. Finally, epidemiological and clinical data connect these processes with health outcomes. This framework transforms exposure assessment from a largely chemical question into a chemical–biological–clinical continuum.

None of these components is new in isolation, and the framework is best understood against the approaches from which it is assembled (Table 1). The exposome concept defines the object of study, the totality of exposures and their biological responses [11,12], but not an inferential sequence for a particular route of exposure. Molecular epidemiology has established a biomarker continuum running from exposure through internal dose, biologically effective dose, and early biological effect to disease [21]. The aggregate exposure pathway organizes exposure from source to target-site concentration and is designed to connect with the adverse outcome pathway, which begins at the molecular initiating event [25,34]; together, these pathways provide a source-to-outcome chain for individual chemicals. The PrecisionTox initiative addresses conserved hazard mechanisms across species (Section 3). What the present framework adds is a specific integration for food: it applies this chain to the dietary exposome and its mixtures, treats interindividual susceptibility as a quantity to be measured rather than a default factor, attaches an explicit evidence requirement to each transition, and links those requirements to the points at which evidence can enter an existing food risk assessment (Section 10).

The five levels were chosen because each corresponds to a distinct level of evidence (Section 2) and answers a distinct question in risk assessment, and the framework becomes operational only when the evidence needed to move between them is specified. Moving from external exposure to an internal dose requires a biomarker validated for the relevant matrix and exposure window, with known toxicokinetics, or a kinetic model calibrated against biomonitoring data [64,65]. Moving from an internal dose to a biological response requires evidence that the biologically effective dose is reached at the target, typically through PBK modeling or in vitro-to-in vivo extrapolation [66], together with a concentration–response relationship in a human-relevant system anchored to a key event in an adverse outcome pathway [67]. Moving from biological response to health outcome requires that the key event or biomarker of effect be linked to an adverse outcome in epidemiological or clinical data [68]. Susceptibility is placed fourth not because it follows the biological response in time but because it is estimated from how the preceding transitions vary between individuals; the evidence it requires is a measured distribution of the modifying factor, kinetic or dynamic, sufficient to replace a default adjustment factor. Section 8 adds the temporal dimension to this sequence, and Section 9 traces one chemical group through every transition.

## 8. Temporal Dimension: Dietary Exposure Across the Life Course

**Figure 2:**
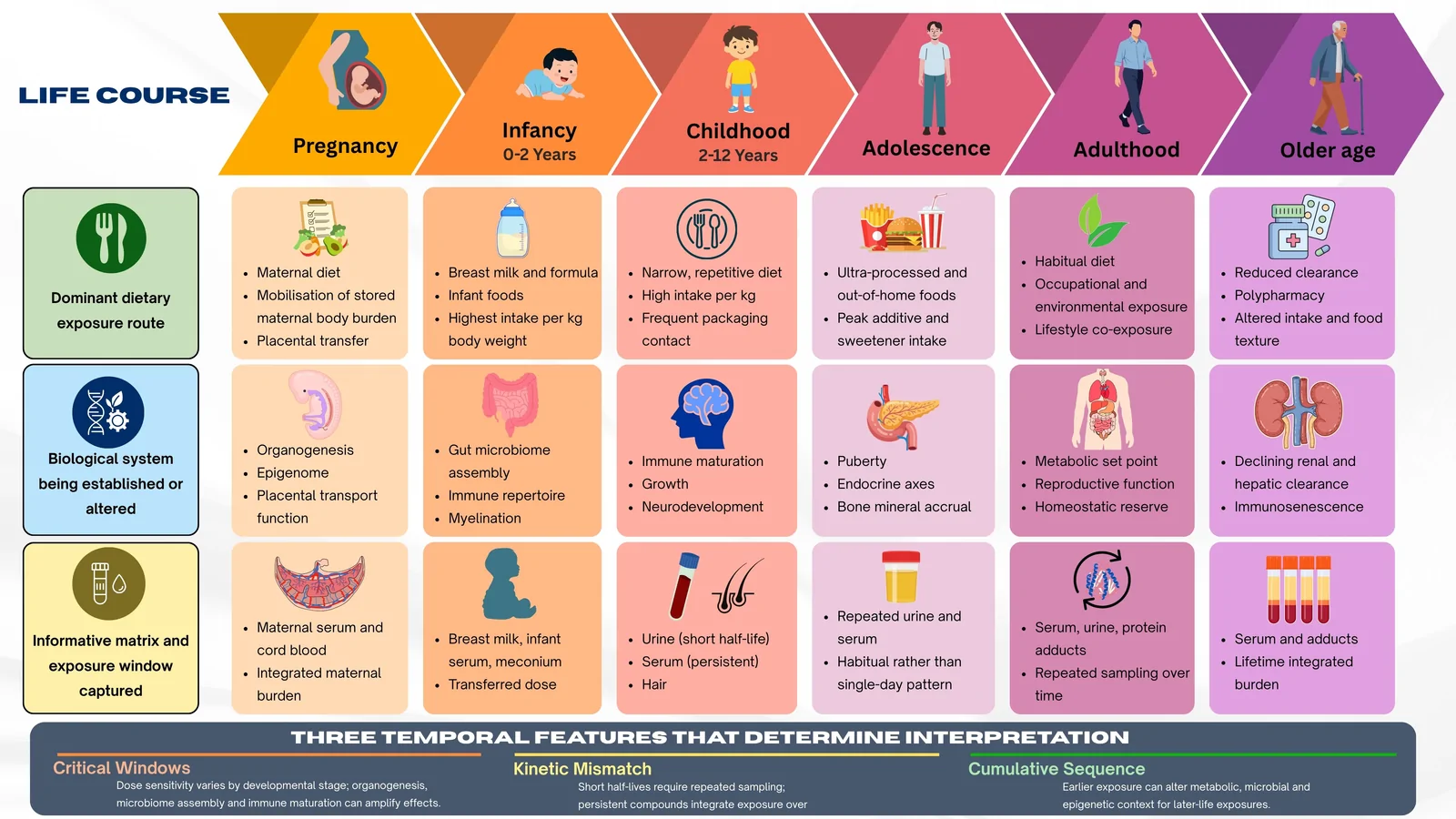
Life course as an axis running through the precision toxicology framework. For each life stage, the figure shows the dominant dietary exposure route, the biological system being established or altered, and the biological matrix that captures the relevant exposure window. The lower panel summarizes the three temporal features that determine how exposure data should be interpreted: critical windows, the mismatch between exposure kinetics and measurement, and the cumulative and sequential nature of exposure.

The framework set out above is most naturally read as a cross-section, that is, as a set of measurements made at one point in time. Dietary exposure is not, however, a cross-sectional quantity: it accumulates, fluctuates, and coincides with developmental stages that differ in sensitivity. Making this temporal dimension explicit is therefore a condition of the framework’s validity rather than an embellishment of it, and we treat the life course as an axis that runs through all five levels rather than as a sixth level appended to them (Figure 2).

Three temporal features are particularly consequential for food safety. The first is the existence of critical windows. In utero and during infancy, the organ systems, immune repertoire, epigenome, and gut microbial community are still being established, and perturbation during these intervals can initiate effects that equivalent exposure in adulthood does not reproduce [16,69]. Placental transfer and lactation are themselves exposure routes, and for persistent compounds, they transfer an accumulated maternal burden rather than a current intake, which is the situation captured by the PFAS assessment discussed in Section 2. The second feature is the mismatch between exposure kinetics and measurement. Chemicals with short biological half-lives, including many plasticizers, preservatives, and current-use pesticides, fluctuate within hours such that a single spot sample may misclassify habitual exposure [70], whereas persistent compounds integrate exposure over years such that a single measurement may reflect exposure that has already ceased. The appropriate sampling design is therefore compound specific, as the aflatoxin example in Section 2 illustrates.

The third feature is cumulative and sequential effects. Exposures are not independent draws: earlier exposure can alter the metabolic, microbial, and epigenetic context in which later exposure is absorbed, transformed, and acted upon, and the biological record of that history is increasingly measurable. In a longitudinal twin study, a measured set of exposures explained roughly a quarter to a third of the variance in epigenetic aging, with much of the remainder attributable to environmental factors not shared between siblings and not captured by the model [71]; this underlines both the promise and the present incompleteness of life-course exposome analysis. For food safety, the operational implications are concrete. Exposure assessment should increasingly be specified with a time base—the window of exposure, the frequency of measurement, and the half-life of the biomarker used—rather than as an undifferentiated annual average; health-based guidance values should indicate the life stage for which they are protective, and cohort designs should favor repeated sampling wherever a compound’s kinetics make a single measurement uninformative.

## 9. From Framework to Practice: A Worked Example for PFAS

A framework is useful only if a real evidence chain can be traced through it. Perfluoroalkyl substances provide some of the most completely documented examples currently available because the major levels of the framework are supported by human biomonitoring and epidemiological, toxicokinetic, and human-relevant mechanistic evidence, and the evidence chain has contributed to enforceable regulation. We therefore use PFAS to illustrate how the five levels and the transitions specified in Section 7 operate together (Table 2).

### 9.1. External Exposure

PFAS reach the consumer through several separately regulated pathways: environmental background contamination of soil and water, and hence of crops and livestock; bioaccumulation in fish and seafood; and migration from fluorinated food-contact materials. Occurrence data from European monitoring identify fish and seafood, eggs, and meat and offal as the main dietary contributors, with locally produced food adding to exposure in contamination hotspots, and European human biomonitoring indicates that a substantial proportion of adolescents have serum concentrations above current health-based guidance values [10]. Validated targeted LC–MS/MS methods quantify the regulated congeners, whereas high-resolution nontarget screening has revealed hundreds of additional PFAS, including replacement compounds, that targeted panels do not include [72].

### 9.2. Internal Dose

Because the serum half-lives of the long-chain PFAS considered here are measured in years, serum concentration integrates exposure over a long period and is a better index of internal dose than any short-term dietary record [28]. Human biomonitoring accordingly provides a quantitative bridge: In the study that supplied the European point of departure (Section 2), plasma PFOA and PFOS concentrations in one-year-old children breastfed for at least four months were markedly higher than those in formula-fed children [27]. Translating between serum concentration and dietary intake requires toxicokinetic modeling, and such models—developed initially for a small number of congeners and now being adapted to less-studied PFAS—allow reverse dosimetry from measured biomarker concentrations back to the intakes that produced them [73].

### 9.3. Biological Response and Mechanistic Validation

An epidemiological association between serum PFAS and an attenuated vaccine antibody response is not by itself sufficient to establish causation or mode of action, and NAMs have been used to strengthen the mechanistic evidence underlying this association. In human peripheral blood mononuclear cell models, PFAS suppress B lymphocyte activation and antibody secretion, and a transcriptomic analysis revealed that glucocorticoid receptor signaling is involved in the mediating pathway; this effect is reversed by a glucocorticoid receptor antagonist, which provides experimentally testable mechanistic support for the epidemiological association [74]. In parallel, transcriptomic benchmark-dose analysis in human liver spheroids, coupled with PBK reverse dosimetry, has been used to derive a candidate tolerable daily intake for PFOA of the same order of magnitude as values derived by conventional means, demonstrating that NAM-derived and epidemiology-derived guidance values can be compared quantitatively rather than only conceptually [75].

### 9.4. Individual Susceptibility

The same dietary intake does not result in the same serum concentration. Elimination differs with physiological state; pregnancy and lactation redistribute the body burden between mothers and infants, and the infant’s burden derives largely from placental and lactational transfer rather than from its own diet [27,28]. Susceptibility in this example is therefore dominated by kinetic rather than dynamic variability, which is itself informative: it identifies where a chemical-specific adjustment factor derived from human toxicokinetic data would most usefully replace a default uncertainty factor [52].

### 9.5. Health Outcomes and Regulatory Translation

As described in Section 2, EFSA derived a tolerable weekly intake of 4.4 ng/kg body weight per week for the sum of PFOA, PFNA, PFHxS, and PFOS from a serum BMDL of 17.5 ng/mL in one-year-old children [28]. Maximum levels of these substances in eggs, fish, crustaceans, mollusks, and meat and offal were subsequently established in Union legislation and are now consolidated in Commission Regulation (EU) 2023/915 [76]. The chain is thus complete: occurrence data identified the contributing foods, biomonitoring established the internal dose, epidemiology identified the outcome, kinetic modeling connected the internal dose to intake, a health-based guidance value was established, and subsequent EU legislation established enforceable maximum levels for PFAS in specified food categories.

Two qualifications should be stated. First, the chain relies on a single immunological critical effect [28]. Second, PFAS are unusually tractable: they are persistent, measurable in a routine matrix, and present at concentrations well above analytical detection limits. For the majority of dietary chemicals—short-lived, present at trace concentrations, and lacking validated biomarkers—no comparable chain currently exists. The example therefore demonstrates that the framework is achievable, not that it is easy; the constraints that separate this case from the general one are the subject of Section 11.

## 10. Regulatory Translation Remains the Critical Test

Precision toxicology will have a limited impact unless its discoveries can ultimately inform regulatory decision-making. Exposomics and nontarget screening should therefore not be positioned as replacements for validated targeted analysis or conventional risk assessment. A more realistic model is a discovery → prioritization → confirmation → mechanistic validation → risk-assessment pipeline. Nontarget exposomics can identify emerging exposure signals, targeted analytical methods can confirm and quantify them, NAMs and mechanistic toxicology can establish biological plausibility, and epidemiological or clinical evidence can determine human relevance. Translation will require standardized sample collection, harmonized analytical workflows, validated reference materials, interoperable databases, transparent bioinformatics, and rigorous characterization of uncertainty [18,35,36].

The principal constraint on this pipeline is therefore not technological availability but regulatory readiness. NAMs and next-generation exposure technologies are becoming increasingly capable, but formal acceptance of a new method relies on the following explicit criteria: a defined purpose and applicability domain, reproducibility within and between laboratories, transferability to laboratories other than the developer’s, performance benchmarked against reference chemicals and existing data, and independent review [77]. Comparability with the legacy datasets that underpin existing health-based guidance values is usually also expected, although whether animal data should serve as the benchmark is itself contested [35,36]. Closing this gap will require regulators, method developers, and the food industry to agree in advance on what constitutes sufficient evidence for a given decision context, since validation and regulatory acceptance of NAMs have proven slow [35,36]. Regulatory translation should therefore become a design criterion for exposomic and NAM studies from their inception rather than an endpoint considered only after scientific discovery.

Translation can be made more concrete by identifying where each type of evidence would enter an existing assessment. Risk assessment of chemicals in food follows an established sequence of hazard identification, hazard characterization, exposure assessment, and risk characterization, with uncertainty analysis running throughout [1,78]. Table 3 maps the components of the proposed framework onto these steps and states, for each, the evidence that would be required before it could influence the outcome. Two features of this mapping are worth noting. The entry requirements are weakest for prioritization, where exposomic screening can already direct monitoring without altering any guidance value, and strongest for hazard characterization, where a NAM-derived point of departure must be benchmarked against the legacy data underpinning the existing value. Exposure assessment is the step at which the framework is closest to routine use because biomonitoring and PBK modeling are already accepted where validated biomarkers exist, as the PFAS example shows.

**Table 3:** Entry points for precision toxicology evidence into food risk assessment. HBGV, health-based guidance value; NAM, new approach methodology; PBK, physiologically based kinetic; POD, point of departure.

Assessment Step	Contribution	Evidence Required Before It Can Influence the Outcome	Current Readiness
Problem formulation and prioritization	Suspect and nontarget screening identifies emerging or unmonitored substances	Reproducible detection and provisional identification; no effect on any guidance value at this stage	Available now
Hazard identification	Omics and NAM screening flag biological activity and candidate modes of action	Concentration–response in a human-relevant system, anchored to a key event; independent replication	Case-by-case
Hazard characterization	NAM-derived POD; mechanistic grouping of chemicals for combined assessment	Defined applicability domain; interlaboratory reproducibility; benchmarking against the legacy data supporting the current HBGV	Not yet routine
Exposure assessment	Human biomonitoring and PBK reverse dosimetry to estimate internal dose and intake	Validated biomarker with known kinetics for the relevant window; representative sampling	Available where biomarkers exist
Uncertainty analysis	Chemical-specific adjustment factors and probabilistic characterization replace default factors	Measured human toxicokinetic or toxicodynamic variability of adequate size and representativeness	Available for well-studied chemicals
Risk characterization and HBGV derivation	Integration of the above into a derived or revised guidance value	Coherent evidence across levels and documented human relevance of the critical effect	Demonstrated for PFAS (Section 9)

The harmonized infrastructure on which these requirements depend is already being built through major European initiatives. The European Human Biomonitoring Initiative (HBM4EU), a joint effort of 30 countries, harmonized human biomonitoring in Europe through common sampling methods, quality control, and data standards for priority chemicals [79]. Its successor, the Partnership for the Assessment of Risks from Chemicals (PARC), launched in 2022 as a seven-year program involving nearly 200 partners, including EFSA, the European Chemicals Agency (ECHA), and the European Environment Agency (EEA), and was designed to integrate human biomonitoring and exposure monitoring with NAM-based hazard assessment for regulatory use [80]. However, a similar integrated system for dietary exposomes that combines harmonized food monitoring, including validated multiresidue methods for cooked foods [81], food consumption data, and human biomonitoring, has not yet been fully established and remains an important gap.

## 11. Limitations and Challenges

The framework described here sets out a direction of travel rather than current capability, and several constraints separate the two. Stating them plainly is a condition of the framework’s credibility, and each has a specific consequence for food safety assessment (Table 4).

### 11.1. Analytical and Metrological Constraints

Nontarget screening generates more features than can be confidently identified, and reported identifications span a range of confidence from exact-mass match to confirmation against an authentic reference standard, only the last of which constitutes a confirmed structure [82]. Quantification without authentic standards adds further uncertainty, and reference materials and spectral libraries remain incomplete for many exposure markers and transformation products [18].

### 11.2. Biomarker Availability and Exposure Misclassification

Validated biomarkers exist for only a small minority of food chemicals. With respect to rapidly metabolized substances, single spot samples yield substantial within-person variability, and the resulting misclassification attenuates exposure–response estimates, so that real associations may be missed [59,70]. Biomarkers also cannot always distinguish dietary from nondietary sources, which limits their use for attributing risk to food specifically [83,84].

### 11.3. Causal Inference in High-Dimensional Data

Exposome-wide analyses test many correlated exposures against correlated outcomes and are vulnerable to false discovery, residual confounding, and reverse causation, particularly in cross-sectional designs [85]. Molecular perturbation can also be adaptive rather than adverse, and separating the two requires mechanistic anchoring, which is often missing [39].

### 11.4. Validation and Regulatory Acceptance

As discussed in Section 10, acceptance requires a defined applicability domain, demonstrated reproducibility and transferability, and benchmarked performance. Discordance between a NAM and a legacy animal study may reflect a limitation of either, and the two are difficult to distinguish retrospectively [35,36].

### 11.5. Mixture Complexity

Empirical interaction data exist for a negligible fraction of realistic combinations. Dose addition within cumulative assessment groups is pragmatic and generally protective, but it remains an assumption whose failure conditions are not well characterized at the exposure levels actually encountered in food [30,31].

### 11.6. Representativeness and Global Equity

Exposomic and biomonitoring capacity and the evidence base it produces are concentrated in high-income countries [15]. Dietary patterns, contaminant profiles, and susceptibility determinants differ substantially elsewhere, so a framework validated only in European and North American populations risks being least informative where the burden is greatest.

### 11.7. Infrastructure, Cost, and Sustainability

Long-term cohorts depend on sustained funding that short funding cycles rarely guarantee [86], and the cost of the repeated biomonitoring measurements needed to limit exposure misclassification forces a trade-off between study size and measurement depth [59].

### 11.8. Ethical and Governance Considerations

Linking dietary, molecular, genetic, and health data at individual resolution raises questions of consent, privacy, data sovereignty, and the return of results of uncertain clinical meaning. Dynamic consent, participatory governance, and federated data stewardship are design requirements rather than downstream safeguards [87].

## 12. Precision Toxicology, Public Health, and Sustainable Food Safety

Precision toxicology also provides a practical pathway toward several United Nations Sustainable Development Goals (SDGs) [88]. Improving the identification and prevention of harmful dietary chemical exposures directly supports SDG 3 (good health and well-being), particularly target 3.9, which seeks to substantially reduce deaths and illnesses from hazardous chemicals and from air, water, and soil pollution and contamination [89]. By 2035, precision food toxicology should aim to establish population-scale dietary exposome cohorts that integrate harmonized food monitoring with repeated human biomonitoring, multiomics, microbiome profiling, and longitudinal health outcomes, building on multicohort exposome designs such as the Human Early Life Exposome (HELIX) study [90]. The field must move from cataloging exposure to quantifying biologically effective doses, from single-chemical thresholds to pathway-based mixture assessment, and from default uncertainty factors toward data-derived adjustment factors that protect identified susceptible subgroups. For this contribution to be equitable, the capacity gap identified in Section 11 must be addressed as part of the agenda rather than after it, a need already described as urgent [15].

## 13. Conclusions and Future Directions

The future of food toxicology should not be framed as a choice between traditional risk assessment and precision toxicology. The two approaches serve different but complementary purposes. Population-based thresholds remain essential for public health protection, whereas exposomics and precision toxicology can reveal exposure patterns, mechanisms, and susceptible populations that conventional approaches may overlook. The major transition for the coming decade may therefore be from measuring chemicals in food to understanding chemical exposures in people. In this transition, the central question will no longer be simply ‘What chemicals are present in food?’ but rather ‘Which exposures reach people, how do they perturb biology, who is most susceptible, and which signals are sufficiently robust to guide prevention and regulation?’ Integrating advanced analytical chemistry, biomonitoring, exposomics, the microbiome, systems toxicology, NAMs, AI, and human health data, precision toxicology could enable earlier identification of emerging hazards, biologically informed assessment of chemical mixtures, and more effective protection of susceptible populations.

Translating this into practice requires agreement on what should be achieved first. We suggest six priorities for the next five to ten years, ordered so that each supports those that follow and corresponds to the actions identified in Table 4.

(i) Validate exposure and effect biomarkers for priority food chemicals, specifying for each the matrix, the exposure window captured, and the half-life, so that measurement can be matched to the question asked. (ii) Harmonize sampling, analytical, and reporting workflows, extending the HBM4EU and PARC models to food monitoring and dietary assessment and expanding reference materials and spectral libraries for transformation products. (iii) Standardize and qualify NAMs against defined regulatory questions, with applicability domains, interlaboratory reproducibility, and benchmarking against the legacy data that support existing guidance values. (iv) Establish external validation and explainability standards for artificial intelligence models used in regulatory contexts, including reporting of the applicability domain and quantified uncertainty. (v) Integrate longitudinal epidemiological and clinical data with repeated biomonitoring so that biological responses can be linked to health outcomes across the life course and extend this capacity beyond high-income settings. (vi) Agree regulatory acceptance criteria in advance, defining for each entry point in Table 3 the evidence that would be sufficient so that studies are designed against that standard rather than assessed against it retrospectively.

Precision toxicology will succeed when chemical detection, biological interpretation, and regulatory action become parts of the same evidence continuum.

## Figures and Tables

**Table 1:** Relation of the proposed framework to existing frameworks. Scope is summarized from the cited sources; the remaining columns give the authors’ assessment. AOP, adverse outcome pathway.

Framework	Scope	Contribution Retained Here	What It Leaves Open	Ref.
Exposome	Totality of exposures across the life course and their biological responses	Multiexposure, life-course perspective	No inferential sequence for a specific route of exposure, and no regulatory endpoint	[11,12]
Biomarker continuum	Exposure → internal dose → biologically effective dose → early effect → disease	Distinction between dose metrics and biological effect	Descriptive; does not specify the evidence required for regulatory use	[21]
Aggregate exposure pathway	Source → external exposure → target-site exposure	Quantitative route from food to target-site concentration	Ends at the biological interface	[25]
AOP	Molecular initiating event → key events → adverse outcome	Mechanistic anchoring of biological response	Chemical-agnostic; does not address dose or who is exposed	[34]
PrecisionTox	Molecular key events conserved across species	Hazard signatures transferable to humans	Does not address dietary exposure or internal dose	[37]
Present framework	Dietary exposure → internal dose → biological response → susceptibility → health outcome	Integration for food; measured susceptibility; evidence stated for each transition; defined regulatory entry points	Demonstrated thus far only for well-characterized chemicals (Section 9 and Section 11)	—

**Table 2:** The five levels of the framework populated for perfluoroalkyl substances. BMDL, benchmark dose lower confidence limit; NAM, new approach methodology; PBK, physiologically based kinetic; TWI, tolerable weekly intake.

Level	Question Addressed	PFAS Evidence	Principal Method	Ref.
1. External exposure	Which foods deliver the exposure?	Seafood, fish, offal, eggs and meat dominate dietary intake; migration from fluorinated food-contact materials; crops and livestock from contaminated land	Targeted LC–MS/MS multiresidue analysis; suspect and nontarget screening for replacements and transformation products	[10,72]
2. Internal dose	What reaches the body, and for how long?	Serum half-lives of years for the long-chain congeners; plasma PFOA and PFOS markedly higher in breastfed than formula-fed one-year-olds; BMDL 17.5 ng/mL in serum of one-year-olds	Human biomonitoring in serum or plasma; PBK and toxicokinetic modeling linking serum concentration to dietary intake	[27,28,73]
3. Biological response	Is the perturbation real, and by what mechanism?	Suppressed B lymphocyte activation and antibody secretion in human cells; glucocorticoid receptor signaling identified and effect reversed by receptor antagonist	Human peripheral blood mononuclear cell NAM of B-cell function; transcriptomic benchmark-dose analysis in liver spheroids	[74,75]
4. Individual susceptibility	Why do equally exposed people differ?	State-dependent elimination; pregnancy and lactation redistribute burden; the infant’s burden derives largely from placental and lactational transfer	Toxicokinetic variability analysis; basis for a chemical-specific adjustment factor in place of a default uncertainty factor	[27,28,52]
5. Health outcome	Does it matter for health, and what follows?	Reduced antibody response to routine vaccination; TWI of 4.4 ng/kg body weight per week for the sum of PFOA, PFNA, PFHxS and PFOS; maximum levels in food	Epidemiological cohorts and clinical endpoints; regulatory derivation and enforcement	[28,76]

**Table 4:** Principal limitations, their consequences for food safety assessment, and the priority action each requires. NAM, new approach methodology.

Domain	Principal Limitation	Consequence for Food Safety Assessment	Priority Action
Analytical	Most nontarget features cannot be identified with regulatory-grade confidence; quantification without authentic standards is uncertain	Detection cannot yet be converted into a defensible exposure estimate for most emerging compounds	Expand reference standards and spectral libraries for food-relevant transformation products; set interlaboratory performance criteria
Biomarkers	Validated biomarkers exist for few dietary chemicals; single spot samples misclassify short half-life compounds	Exposure–response relationships are attenuated, so real effects may be reported as absent	Develop and validate biomarkers for priority food chemicals; use repeated sampling where kinetics require it
Causal inference	High-dimensional analysis is vulnerable to false discovery, confounding and reverse causation; adaptive and adverse responses are hard to separate	Associations may not support regulatory action even when statistically robust	Require mechanistic anchoring and independent replication before a signal enters an assessment; prespecify analysis plans
Validation and acceptance	Acceptance requires defined applicability domains, reproducibility, transferability and benchmarked performance	Mechanistically superior evidence may be excluded from decisions that continue to rely on legacy data	Agree evidentiary sufficiency with regulators in advance; treat regulatory translation as a design criterion from study inception
Mixtures	Interaction data exist for a negligible fraction of realistic combinations; dose addition is an assumption of unknown robustness at dietary levels	Combined exposure is assessed on a default that has not been tested where it matters	Test dose additivity at realistic concentrations; refine cumulative assessment groups on mechanistic rather than structural grounds
Representativeness	Capacity and published evidence are concentrated in high-income countries	Guidance is least informative where the dietary chemical burden is greatest	Build analytical and cohort capacity in low- and middle-income settings as part of the agenda
Infrastructure and cost	Cohort timescales exceed funding cycles; study size and measurement depth compete for the same resources	Longitudinal evidence of the required quality may never be assembled	Establish long-horizon funding, harmonized collection protocols, interoperable data standards and sustained biobanking
Ethics and governance	Individual-resolution linkage raises consent, privacy, data sovereignty and result-return questions	Participation and public trust may be lost, and individual findings may cause harm without actionable interpretation	Adopt dynamic consent, participatory governance and federated data stewardship by design; define result-return policy before data collection

## Data Availability

No new data were created or analyzed in this study. Data sharing is not applicable to this article.

## Abbreviations

The following abbreviations are used in this manuscript:

AI	Artificial intelligence
AOP	Adverse outcome pathway
BMDL	Benchmark dose lower confidence limit
CYP450	Cytochrome P450
ECHA	European Chemicals Agency
EEA	European Environment Agency
EFSA	European Food Safety Authority
GC–MS/MS	Gas chromatography–tandem mass spectrometry
HBM4EU	European Human Biomonitoring Initiative
HELIX	Human Early Life Exposome
JECFA	Joint FAO/WHO Expert Committee on Food Additives
LC–MS/MS	Liquid chromatography–tandem mass spectrometry
NAM	New approach methodology
PARC	Partnership for the Assessment of Risks from Chemicals
PBK	Physiologically based kinetic
PFAS	Per- and polyfluoroalkyl substances
PFHxS	Perfluorohexanesulfonic acid
PFNA	Perfluorononanoic acid
PFOA	Perfluorooctanoic acid
PFOS	Perfluorooctanesulfonic acid
QTOF–MS	Quadrupole time-of-flight mass spectrometry
SDG	Sustainable Development Goal
WHO	World Health Organization
